# Diffusion Tensor Imaging Study of White Matter Damage in Chronic Meningitis

**DOI:** 10.1371/journal.pone.0098210

**Published:** 2014-06-03

**Authors:** Wei-Che Lin, Pei-Chin Chen, Hung-Chen Wang, Nai-Wen Tsai, Kun-Hsien Chou, Hsiu-Ling Chen, Yu-Jih Su, Ching-Po Lin, Shau-Hsuan Li, Wen-Neng Chang, Cheng-Hsien Lu

**Affiliations:** 1 Department of Radiology, Kaohsiung Chang Gung Memorial Hospital, Chang Gung University College of Medicine, Kaohsiung, Taiwan; 2 Department of Neurosurgery, Kaohsiung Chang Gung Memorial Hospital, Chang Gung University College of Medicine, Kaohsiung, Taiwan; 3 Department of Neurology, Kaohsiung Chang Gung Memorial Hospital, Chang Gung University College of Medicine, Kaohsiung, Taiwan; 4 Institute of Neuroscience, National Yang-Ming University, Taipei, Taiwan; 5 Internal Medicine, Kaohsiung Chang Gung Memorial Hospital, Chang Gung University College of Medicine, Kaohsiung, Taiwan; 6 Department of Biological Science, National Sun Yat-Sen University, Kaohsiung, Taiwan; University of Pécs Medical School, Hungary

## Abstract

Tuberculous meningitis (TBM) and cryptococcal meningitis (CM) are two of the most common types of chronic meningitis. This study aimed to assess whether chronic neuro-psychological sequelae are associated with micro-structure white matter (WM) damage in HIV-negative chronic meningitis. Nineteen HIV-negative TBM patients, 13 HIV-negative CM patients, and 32 sex- and age-matched healthy volunteers were evaluated and compared. The clinical relevance of WM integrity was studied using voxel-based diffusion tensor imaging (DTI) magnetic resonance imaging. All of the participants underwent complete medical and neurologic examinations, and neuro-psychological testing. Differences in DTI indices correlated with the presence of neuro-psychological rating scores and cerebrospinal fluid (CSF) analysis during the initial hospitalization. Patients with CM had more severe cognitive deficits than healthy subjects, especially in TBM. There were changes in WM integrity in several limbic regions, including the para-hippocampal gyrus and cingulate gyrus, and in the WM close to the globus pallidus. A decline in WM integrity close to the globus pallidus and anterior cingulate gyrus was associated with worse CSF analysis profiles. Poorer DTI parameters directly correlated with worse cognitive performance on follow-up. These correlations suggest that WM alterations may be involved in the psychopathology and pathophysiology of co-morbidities. Abnormalities in the limbic system and globus pallidus, with their close relationship to the CSF space, may be specific biomarkers for disease evaluation.

## Introduction

Tuberculous meningitis (TBM) and cryptococcal meningitis (CM) are two of the most common types of chronic meningitis. They have similar clinical presentations and cerebrospinal fluid (CSF) features and despite the advent of new antimicrobial therapies, their morbidity and mortality remain high. The high rate of neurologic sequelae among survivors indicates that therapy is far from being satisfactory [1]–[3]. Moreover, detailed neuro-psychological evaluation to detect cognitive sequelae after complete treatment of chronic meningitis [4]–[6] or in co-morbidity with HIV infection [7] is limited.

In the diagnosis of chronic meningitis, magnetic resonance imaging (MRI) provided greater inherent sensitivity and specificity than CT scan. Advanced MRI techniques, such as magnetization transfer imaging, diffusion imaging, and proton magnetic resonance spectroscopy may also provide better tissue characterization in CNS chronic meningitis [6], [8]. Diffusion tensor imaging (DTI) is a non-invasive technique that can explore and provide evidence of micro-structural features of WM that can be closely correlated with differences in cognitive functions [9]–[12]. It can also quantify peri-ventricular white matter (WM) changes in neonatal meningitis and suggest that patients with abnormal outcome have decreased anisotropy values [13]. A small pilot study has revealed significant WM ultra-structural damage in CM using DTI in multiple selected regions of interest, including the corpus callosum, peri-ventricular WM, and lentiform nucleus [6]. Higher CSF cryptococcal-antigen titer on admission is further associated with unfavorable DTI parameters. Although various causes have been proposed, hydrocephalus or high microbial CSF burden causing direct or indirect damage to vulnerable anatomical sites is regarded as the most likely explanation [6]. However, manual analysis of regions of interest in a spectrum of meningitis-related abnormalities based on previous reports may overlook and underestimate injury to the global brain parenchyma from meningitis.

The limbic system consists of the phylogenetically old limbic system and other sub-cortical structures and their connections that have direct contact with the CSF system. Injury may cause neuro-psychological impairment in attention, memory, and emotions, but whether or not the limbic system may suffer from more damage than the neo-cortex and their association with the clinical sequelae of chronic meningitis remains unknown. Increasing the understanding of cognitive impairment in such patients may help improve treatment strategies.

Whole-brain voxel-based morphometry (VBM) analysis of DTI is an operator-independent approach that allows for the analysis of entire brain volumes without *a priori* hypothesis regarding the anatomic location of between-group differences [14]. Among the DTI indices, the MD (average diffusion coefficient, [(λ1+λ2+λ3)/3] is recognized as an isotropic diffusion with free movement of water and an index of alterations in brain micro-structures. Axial diffusivity (AD) is the diffusion coefficient along the direction of maximal “apparent” diffusion (principal diffusion component, λ1). The second and third eigenvalues in the DTI can be averaged and presented as radial diffusivity (RD) (transverse diffusion component, [(λ2+λ3)/2]). Lastly, the relative ratio of axial to radial diffusivities is known as FA, indicating the integrity of white matter fibers [15], [16].

To comprehensively explore the different types of diffusion changes in chronic meningitis, brain integrity in this study was measured by four diffusivity indices: MD, FA, RD, and AD. The present study targeted brain micro-structure integrity and aimed to explore the psychopathology and pathophysiology of co-morbidities in chronic meningitis. First, CSF examination, cognition functions, and the effects of meningitis on the brain were investigated between chronic meningitis subjects and healthy controls using VBM analysis of DTI. Second, micro-structure differences from direct group comparisons associated with the initial CSF examination and cognition decline during long-term follow-up were determined in HIV-negative chronic meningitis patients.

## Patients and Methods

### Inclusion Criteria

Chang Gung Memorial Hospital's Institutional Review Committee on Human Research approved the study and all patients provided written informed consent. From January 2009 to December 2010, 24 HIV-negative TBM and 20 HIV-negative CM patients who had discontinued anti-TB and anti-fungal therapy and had been discharged from the hospital for more than three months were seen at the Outpatient Neurology Clinic.

### Diagnostic Criteria

This is an extension of a prior study [6]. The diagnostic criteria for tuberculous and cryptococcal meningitis were according to previously published data [6], [17]. Tuberculous meningitis (TBM) was defined as: (1) isolation of Mycobacterium tuberculosis (M. tuberculosis) in one or more CSF cultures and/or positive polymerase chain reaction with clinical features of chronic meningitis; or (2) isolation of M. tuberculosis from outside the central nervous system, with clinical presentations of chronic meningitis, and typical CSF features, including pleocytosis with >20 cells, predominantly lymphocytes (>60%), protein >100 mg%, glucose <60% of corresponding blood glucose, and negative India ink studies and cytology for malignant cells [17].

Cryptococcal meningitis (CM) was defined as: (1) isolation of *Cryptococcus neoformans* in one or more CSF cultures, positive CSF cryptococcal antigen titer, or positive CSF India ink test and clinical features of meningitis; or (2) isolation of *C. neoformans* in blood culture with clinical presentations of meningitis and typical CSF features [6].

### Exclusion Criteria

Patients were excluded if they had any of the following: 1) age <20 years or >75 years; 2) evidence for alcoholism or any other addictive disorders, or known affective or psychiatric diseases other than that caused by sedatives or neuroleptics; and 3) known neurologic disorders potentially affecting the central nervous system, or severe recent life events that may interfere with neuro-psychological testing. There were 44 patients with meningitis, including 19 TBM and 13 CM patients, who were enrolled and received both neuro-imaging and neuro-psychological follow-up examination. Twelve patients were excluded, including seven with severe neurologic sequelae and five with poor neuro-imaging quality.

### Conventional Imaging

Cranial computed tomography (CT) scans and/or MRI studies were done on admission and repeated if there was clinical deterioration before discharge. The serial imaging studies were collected and analyzed by neuro-radiologists experienced in the field of central nervous system (CNS) infections. Brain lesions were recorded following a pre-established check-list and chronic meningitis-related lesions were defined by any of the following: meningeal enhancement, basal ganglia infarction/dilated Virchow-Robin spaces, cerebritis, and hydrocephalus. Cerebritis was diagnosed by the presence of focal hypo-intensity on T1, hyper-intensity on T2, and small areas of patchy enhancement on post-contrast scan [18].

Hydrocephalus was diagnosed by the presence of a dilated temporal horn of the lateral ventricle without obvious brain atrophy and/or an Evan's ratio (the ratio of the ventricular width of the bilateral frontal horn to the maximum bi-parietal diameter) >0.3 on CT or MRI during admission [19]. Patients with hydrocephalus and evidence of increased intracranial pressure or clinical deterioration underwent ventriculo-peritoneal shunting. Information on the Glasgow coma scale (GCS) was obtained at the time of the recording. All of the patients underwent complete medical and neurologic examinations, and neuro-psychological testing. Neurologists integrated the clinical manifestations and neuro-psychological findings.

For comparison, 32 sex- and age-matched healthy subjects without a medical history of neurologic disease and with similar lengths of school education were recruited and served as the control group.

### Neuro-psychological Testing

A clinical psychologist blinded to the patients' exposure status performed the neuro-psychological battery of tests, which focused on attention, execution, speech and language, and amnesic and visuo-construction function. Attention functions were measured by digit span score from the Wechsler Adult Intelligence scale-III (WAIS-III) [20] and attention and orientation score from the Cognitive Ability Screening Instrument (CASI) [21]. Executive functions were measured using digit symbol coding, similarity, arithmetic, picture arrangement, and matrix reasoning scores from WAIS-III [20]; abstract thinking scores from CASI [21]; and concept, errors, and perseveration scores from the Wisconsin Card Sorting Test, WCST-64 (Computer Version Scoring Program) [22].

Memory functions were measured using short- and long-term memory scores from CASI [21] and information scores from WAIS-III [20]. Speech and language ability were measured using vocabulary and comprehension scores from WAIS-III [20], and language and semantic fluency scores from CASI [21].

Visuo-construction ability was assessed using the score of picture completion and block design from WAIS-III, and the drawing score from CASI [21]. The Beck Depression Inventory II (BDI) and Beck Anxiety Inventory (BAI) were 21-item self-report questionnaires used to evaluate the severity of depression [23].

### Image Acquisition

The MR data were acquired on a 3.0T whole body GE Signa MRI system (General Electric Healthcare, Milwaukee, WI, USA). To minimize motion artifacts generated during the scan, the subject's head was immobilized with foam pillows inside the coil. The T1-weighted structured images were acquired parallel to the anterior-posterior commissure (AC-PC) through the whole head using the 3D-FSPGR sequence [repetition time (TR) = 9.492 ms, echo time (TE) = 3.888 ms, flip angle 20°, field of view (FOV) = 24×24 cm, matrix size = 512×512, 110 continuous slices with the slice thickness of 1.3 mm and in-plane spatial resolution of 0.47×0.47 mm) to aid the localization of fractional anisotropy differences.

The DTI were acquired for whole-brain voxel-wise analysis on the WM micro-structure by using a single-shot echo-planar imaging sequence (TR = 15800 ms, TE = 77 ms, number of excitation (NEX)  = 3, matrix size = 128×128, field of view (FOV) = 25.6 cm, voxel size = 2×2×2.5 mm^3^, 55 axial oblique slices without gaps). The DTI gradient encoding schemes included 13 non-collinear directions with a b-value of 1000 s/mm^2^ and a non-diffusion weighted image volume (null image, b-value 0 s/mm^2^).

### Data Pre-processing

The FA maps for each subject were computed using an in-house program registered to the ICBM 152 template (Montreal Neurological Institute). First, to reduce the error term due to image registration and bias in template selection, a specific customized group template was created for the study. This involved spatially normalizing each structural MR image to the ICBM 152 template using the optimum 12-parameter affine transformation. All of the normalized T1W images were then averaged and smoothened with an isotropic 8 mm full-width at half maximum Gaussian kernel, thereby creating the customized template.

Second, non-diffusion weighted (b = 0) images of an individual subject were co-registered to their T1W images as the cost function based on normalized mutual information. The registration parameters were subsequently applied on the FA maps that were inherently registered to other diffusion-weighted images during the acquisition. These FA maps were also skull-stripped to remove non-brain tissue and background noise by utilizing the Brain Extraction Tool (BET) compiled in the FSL library 4.1 (Oxford Centre for Functional Magnetic Resonance Imaging of the Brain, Oxford University, Oxford, UK).

Third, all of the 64 T1W scans were transformed to the same stereotactic space as the customized template image by applying an affine transformation with 12 degrees of freedom together with a series of non-linear warps characterized by a linear combination of three dimension discrete cosine transform (DCT) basis functions. The transformation parameters derived from this step were also applied to the FA maps, which were then effectively registered to the MNI space.

### Statistical Analysis

#### Baseline Clinical Characteristics between Groups

Clinical data and educational information were compared by one-way analysis of variance (ANOVA). The GCS on initial admission and on discharge between the two patient groups were analyzed by Wilcoxon rank sum test. Sex and the use of ventriculo – peritoneal shunt between groups were analyzed by Chi-square test or Fisher's exact test, as appropriate. The Mann-Whitney test was used to analyze the CSF examination, including white cell count, lactate, protein and glucose levels, and CSF/serum glucose ratio. Statistical differences in NP tests among the groups were estimated by one-way analysis of covariance (ANCOVA) with age, sex, and educational level as covariates. Post-hoc analysis was performed with Bonferroni test. Statistical significance was set at *p*<0.05.

#### Analysis of Group Comparison on FA Maps

All image processing, including image registration, spatial normalization, customized template creation, and voxel-wise statistical comparisons, were manipulated using the Statistical Parametric Mapping 8 (SPM8) (Wellcome Department of Cognitive Neurology, London, UK) in MATLAB 7.8.0 (MathWorks, MA). Voxel-based analysis on WM area was performed with SPM8 to investigate FA differences among the groups [24]. First, differences in the FA maps between the 32 chronic meningitis patients (TBM and CM) and the controls were compared.

Second, differences in the FA maps among the three groups – the TBM group/normal control group, the CM group/normal control group, and the TBM group/CM group – were also compared. Analysis of covariance (ANCOVA) was performed with age and sex as covariates to investigate FA differences between groups. In post-hoc tests, six contrasts were used to detect where each voxel had a higher or lower fractional anisotropy when comparing two of the three groups.

Since DTI was sensitive to WM alterations, a customized WM mask threshold at 0.2 was used as an explicit mask to successfully exclude voxels, which consisted of grey matter or cerebral spinal fluid in the majority of subjects. The FA differences were significant at the individual voxel level at *p*<0.001 and the extended cluster size >20 voxels.

After the initial VBM analysis, all of the FA value differences between groups based on the Johns Hopkins University DTI-based WM atlas, which is included in FSL atlas tool (http://fsl.fmrib.ox.ac.uk/fsl/fslwiki/Atlases), were reported. To identify the significant WM clusters that corresponded to gray matter areas, the GingleALE toolbox (The BrainMap Development Team; http://brainmap.org/ale/index.html) and Talairach and Tournoux atlas (http://www.talairach.org/index.html) were used.

#### Correlation between Regional DTI-related Indices and Clinical Evaluations

Partial Pearson correlation analysis with age, sex, and years of formal education as nuisance covariates were performed to correlate the clinical evaluations (i.e., GCS, CSF study during admission, and cognitive function on follow-up) with the regional DTI-related indices within the patient groups. Statistical significance was set at *p*<0.05. All statistical analyses were performed using the SPSS software, version 10.0 (SPSS Inc, Chicago, IL).

## Results

### Baseline Clinical Characteristics between Groups

The baseline clinical characteristics, neuro-imaging findings, and cognitive function of all subjects were listed in Table 1. The TBM and CM patients were followed-up for a median of 81 months (range, 26–108 months) and 115 months (range, 42–125 months), respectively (*p* = 0.74). Statistical analysis of the clinical manifestations and neuro-imaging findings between patient groups were significant for CSF glucose level (*p* = 0.018), CSF/serum glucose ratio (*p* = 0.030), and VP shunt use. There was no significant difference in MRI findings between the acute phase and follow-up.

**Table 1:** Demographic data and neuro-psychological assessments of the tuberculous meningitis (TBM), cryptococcal meningitis (CM), and normal control groups.

Demographic Data	Normal control (n = 32)	TBM (n = 19)	CM (n = 13)	Total chronic meningitis (TBM and CM)	F_1_ or X_1_^2^	*p*_1_ value	F_2_ or X_2_^2^	*p*_2_ value
Age at follow-up	50.5±15.3	51.8±18.5	52.0±13.3	51.9±16.4	0.063	0.939	0.007	0.931
Sex (Male/Female)	27M/5F	15M/4F	12M/1F	27M/5F	0.499	0.593	0.000	1.000
Years of Education	12.6±4.5	9.2±4.9	11.6±4.8	10.2±4.8	3.368	0.061	2.738	0.103
Glasgow Coma Scale at admission	-	14.4±1.5	13.5±2.9	14.0±2.2	3.745	0.236	-	-
Glasgow Coma Scale at discharge	-	14.9±0.5	14.2±2.2	14.6±1.48	6.914	0.259	-	-
Duration of follow-up (months)α	-	81 (26, 108)	115 (42, 125)	69 (28, 122)		0. 74	-	-
CSF White cell count (/mm^3^)α	-	104 (21, 140)	144 (34, 240)	109 (38, 219)		0.651	-	-
CSF Protein (mg/dL)α	-	129 (75, 210)	143 (93, 251)	129 (87, 210)		0.761	-	-
CSF Lactate (mg/dL)α	-	33 (17, 50)	25 (18, 49)	29 (18, 51)		0.700	-	-
CSF Glucose (mg/dL)α	-	52 (36, 78)	37 (15, 49)	49 (28, 59)		**0.018**	-	-
CSF/Serum glucose ratioα	-	0.40 (0.23, 0.50)	0.32 (0.06, 0.37)	0.35 (0.21, 0.47)		**0.030**	-	-
Hydrocephalus with V-P shunting procedure	-	3	7	10		**0.047**	-	-
**MRI imaging during acute phase**								
Meningeal enhancement	-	12	6	18	0.907	0.341		
BG infarction/V-R s paces dilatation	-	7	6	13	0.277	0.598		
Cerebritis	-	8	2	10	2.565	0.109		
Hydrocephalus	-	2	7	9	1.758	0.185		
**MRI imaging during follow-up**								
Meningeal enhancement	-	2	0		3.118	0.157		
BG infarction/V-R s paces dilatation	-	0	0		-	-		
Cerebritis	-	3	1		2.239	0.279		
Hydrocephalus	-	4	7		0.126	1.000		
**Neuro-psychological Assessments**								
**Attention Function**								
Digit span	11.42 (2.77)	8.50±3.78	9.54±3.15	8.94±3.5	2.430	0.098	**6.008**	**0.017**
Attention	7.78±0.57	6.84±0.29	7.48±0.33	7.00±1.65	2.425	0.098	2.477	0.121
Orientation	17.93±0.26	15.79±4.40	16.92±2.18	16.25±3.66	1.860	0.165	**4.585**	**0.036**
**Executive Function**								
Digit symbol coding	11.54±2.92^#^ §	6.94±2.86#	9.08±3.43§	7.84±3.24	9.655	**<0.000**	**17.636**	**<0.001**
Similarity	11.46±2.33#	7.84±3.24#	9.62±3.36	8.56±3.35	6.222	**0.004**	**11.697**	**0.001**
Arithmetic	10.82±2.51	8.06±2.69	10.08±3.28	8.90±3.07	2.419	0.099	**4.970**	**0.030**
Picture arrangement	11.46±3.34#	7.94±3.39#	9.77±3.00	8.71±3.31	4.760	**0.012**	**7.981**	**0.006**
Matrix reasoning	10.61±3.35	8.21±3.77	9.54±2.99	8.75±3.48	0.846	0.435	1.949	0.168
Abstract thinking	10.75±1.32	9.47±2.65	10.08±2.14	9.72±2.44	1.238	0.298	1.701	0.197
Mental Manipulation	9.25±1.14	7.84±3.04	8.85±1.72	8.25±2.60	0.805	0.452	1.421	0.238
Concept from WCSTα	51.22±28.23	37.75±32.04	50.48±30.89	42.9±31.72	1.039	0.360	0.612	0.437
Non-perseveration error from WCSTα	13.56±10.01	18.00±14.54	13.92±11.67	16.34±13.40	0.429	0.653	0.303	0.584
Perseveration error from WCSTα	10.84±5.01	13.21±11.75	13.32±7.57	13.26±10.11	0.541	0.585	1.086	0.302
Perseveration response from WCSTα	12.16±5.86	15.47±13.26	14.62±9.37	15.13±13.22	0.537	0.588	1.038	0.312
**Memory Function**								
Short term memory	10.58±2.02	8.52±3.77	9.35±2.60	8.86±3.32	1.716	0.190	3.146	0.081
Long term memory	9.86±0.54	9.26±1.66	9.85±0.55	9.50±1.34	0.924	0.403	1.189	0.280
Information	11.29±3.41	8.42±2.43	9.54±2.63	8.88±2.54	2.107	0.132	**6.657**	**0.012**
**Speech and Language**								
Vocabulary	11.39±3.11	9.05±3.26	10.54±2.93	9.66±3.17	0.424	0.657	1.187	0.280
Comprehension	12.21±3.14	8.79±3.28	10.15±3.51	9.34±3.39	2.553	0.086	**6.069**	**0.017**
Language	9.97±0.11	9.80±0.43	9.31±1.13	9.51±0.94	0.894	0.415	0.550	0.461
Semantic fluency	8.68±1.70	7.47±2.57	8.00±1.68	7.69±2.24	0.878	0.421	1.364	0.248
**Visuo-construction Function**								
Picture Complete	10.57±3.46#	7.05±3.37#	8.69±2.59	7.72±3.14	5.354	**0.007**	**10.136**	**0.002**
Block design	11.50±3.12^#^ §	8.42±2.81#	9.15±3.89§	8.72±3.26	5.383	**0.007**	**10.937**	**0.002**
Drawing	9.96±0.19	8.53±3.15	9.23±1.36	8.81±2.57	1.823	0.171	**4.483**	**0.038**
**Beck Depression Inventory**	6.88±6.52	10.05±9.19	11.23±10.90	10.53±10.21	0.676	0.513	1.491	0.227

Data are presented as mean ± SD.

α Median (inter-quartile range) value.

F_1_, X_1_^2^ and p_1_ represent comparisons among the TBM, CM, and control groups.

F_2_, X_2_^2^ and p_2_ represent comparison between the controls and all chronic meningitis patients in the study.

# Significant difference between the control and TBM groups in post-hoc analysis with Bonferroni's correction.

§ Significant difference between the control and CM groups in post-hoc analysis with Bonferroni's correction.

Boldfaced values represent significant differences (*p≤*0.05).

In the two-group analyses, chronic meningitis had worse cognitive examination, including attention, execution, memory, speech, language, and visuo-construction function. In three-group analysis, executive function [*Digit symbol coding* (F(2, 58) = 9.655; *p*<0.001), *similarity* (F(2, 58) = 6.222; *p* = 0.004), *picture arrangement* (F(2, 58) = 4.760; *p* = 0.012)] and visuo-construction function [*Picture Complete* (F(2, 58) = 5.354; *p* = 0.007), and *block design*, (F(2, 58) = 5.383; *p* = 0.007)] were worse in TBM patients than in normal controls. Executive function [*Digit symbol coding*] and visuo-construction function [*Block Design*] were also worse in CM patients than in normal controls. There were no significant differences in NP tests between the two groups.

### Group Comparisons on FA Maps

**Figure 1:**
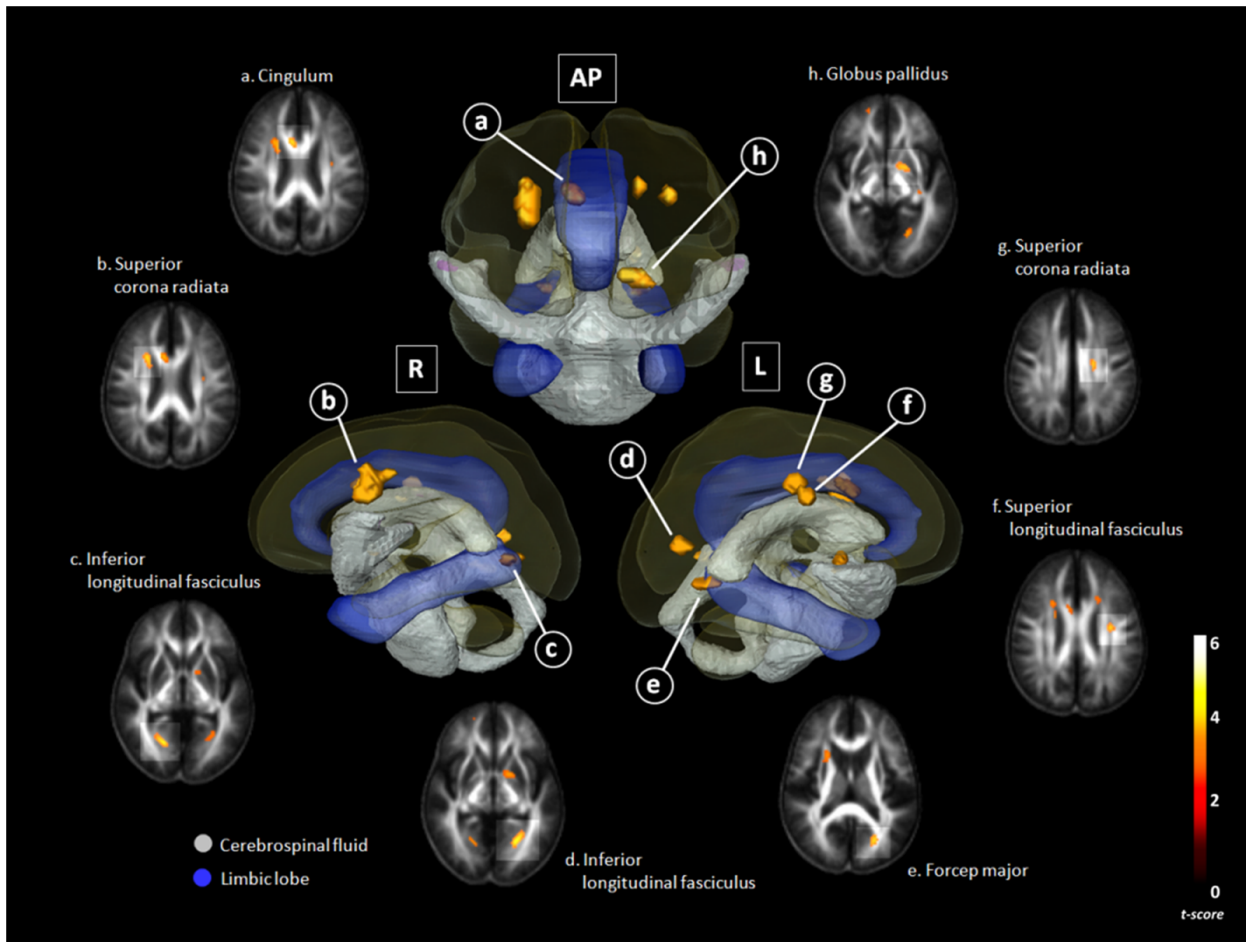
Comparison of fractional anisotropy (FA) between chronic meningitis patients and healthy controls . There was a close relationship between Diffusion Tensor Imaging (DTI) deficits in the limbic system and basal ganglia, and their surrounding CSF space. The correlation between FA decline and CSF space were shown in three-dimensional configuration of the right lateral view (R), antero-posterior view (AP), and left lateral view (L). Gray color, the ventricular system and deep brain arachnoid CSF space; Blue color, parts of the limbic system (cingulate gyrus and para-hippocampal gyrus); Yellow voxels, regions with significantly lower FA value in chronic meningitis vs. normal control (*p*<0.001, corrected).

The chronic meningitis group had significantly lower FA in several WM regions than the control group. Together with decreased FA, there were WMs with increased RD, and small or no decreased AD in the para-hippocampus (bilateral inferior longitudinal fasciculus), cingulate gyrus (right cingulum and bilateral superior corona radiate), left pre-central gyrus (superior longitudinal fasciculus), and left globus pallidus. Together with decreased FA, there were WMs with increased RD and decreased AD in the left cuneus (forceps major) (Table 2; Fig. 1).

**Table 2:** Regions showing lower fractional anisotropy (FA) values in HIV-negative chronic meningitis (n = 32) than in normal controls (n = 32).

MNI atlas coordinates			Voxel size	White matter tract	Nearest Grey MatterΨ	FA mean (SD)		*t_max_*	Diffusivity values (Meningitis-Normal)		
X	Y	Z				Normal	Meningitis		MD	AD	RD
−22	−62	−2	29	Left Inferior Longitudinal Fasciculus	Para-hippocampal Gyrus, BA19	0.34 (0.08)	0.26 (0.07)	4.17	44.5	−28.4	81*
20	−64	0	31	Right Inferior Longitudinal Fasciculus	Para-hippocampal Gyrus, BA 19	0.35 (0.07)	0.28 (0.08)	4.11	44.3*	−13.6	73.2*
8	16	28	37	Right Cingulum	Cingulate Gyrus, BA24	0.55 (0.10)	0.47 (0.12)	3.82	170.5	82.2	214.7*
24	14	28	110	Right Superior Corona Radiata	Cingulate Gyrus, BA32	0.41 (0.04)	0.37 (0.04)	3.82	67.6*	45.9	78.5*
−22	−70	16	27	Left Forceps Major	Cuneus, BA30	0.45 (0.11)	0.35 (0.16)	3.76	34.7	−76.3*	90.2*
−32	−8	32	21	Left Superior Longitudinal Fasciculus	Pre-central Gyrus, BA 6	0.32 (0.05)	0.28 (0.04)	3.73	39.4	14.3	51.9*
−20	−12	38	21	Left Superior Corona Radiata	Cingulate Gyrus, BA 24	0.35 (0.05)	0.31 (0.04)	3.72	127.6	116.2	133.3*
−18	4	−4	29	WM close to Left Globus Pallidus	Lentiform Nucleus	0.38 (0.07)	0.31 (0.06)	3.62	42.9	−19.7	74.3*

The diffusivity values describe differences (meningitis vs. normal) in mean diffusivity (MD), axial diffusivity (AD), and radial diffusivities (RD) (mm^2^/s) multiplied by 10^−6^.

*Significant differences among MD, AD, and RD were adjusted with age, sex, and education as covariates (*p*≤0.05).

Ψ Nearest Gray Matter was near the center of the 5-mm radius search area.

Abbreviations: MNI, Montreal Neurological Institute; BA, Brodmann area.

**Figure 2:**
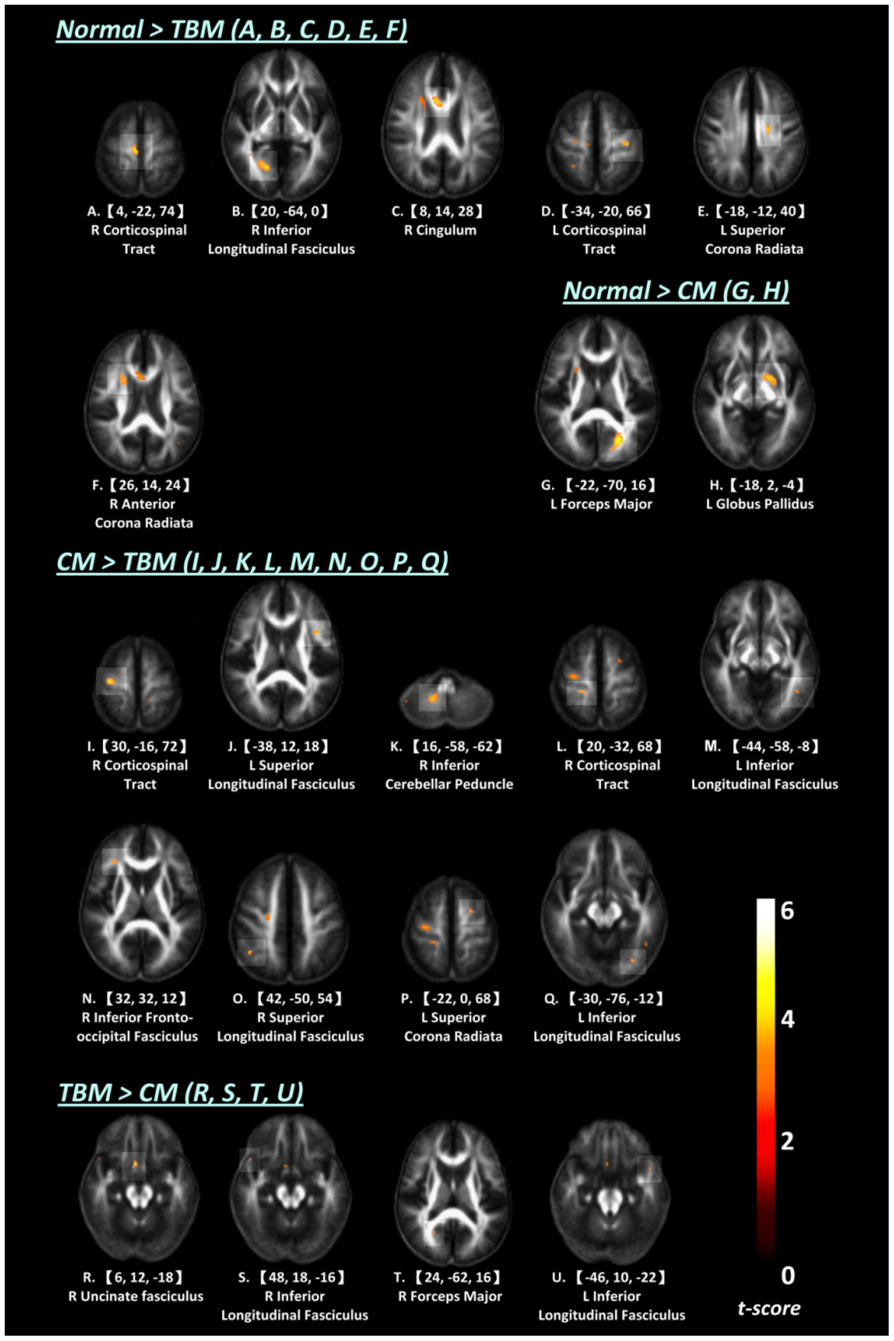
Comparisons of fractional anisotropy (FA) between groups. (**A–F**) Regions with significantly lower FA value in TBM vs. controls. (**G–H**) Regions with significantly lower FA value in CM vs. controls. (**I–Q**) Regions with significantly lower FA value in TBM vs. CM. (**R–U**) Regions with significantly lower FA value in CM vs. TBM.

Differences in regional WM integrity in the FA maps between TBM and controls, between CM and controls, and between TBM and CM patients were shown in Figure 2 and Table S1.

### Correlation between Regional DTI-Related Indices and Clinical Evaluations

#### DTI Indices and Baseline Clinical Characteristics

**Figure 3:**
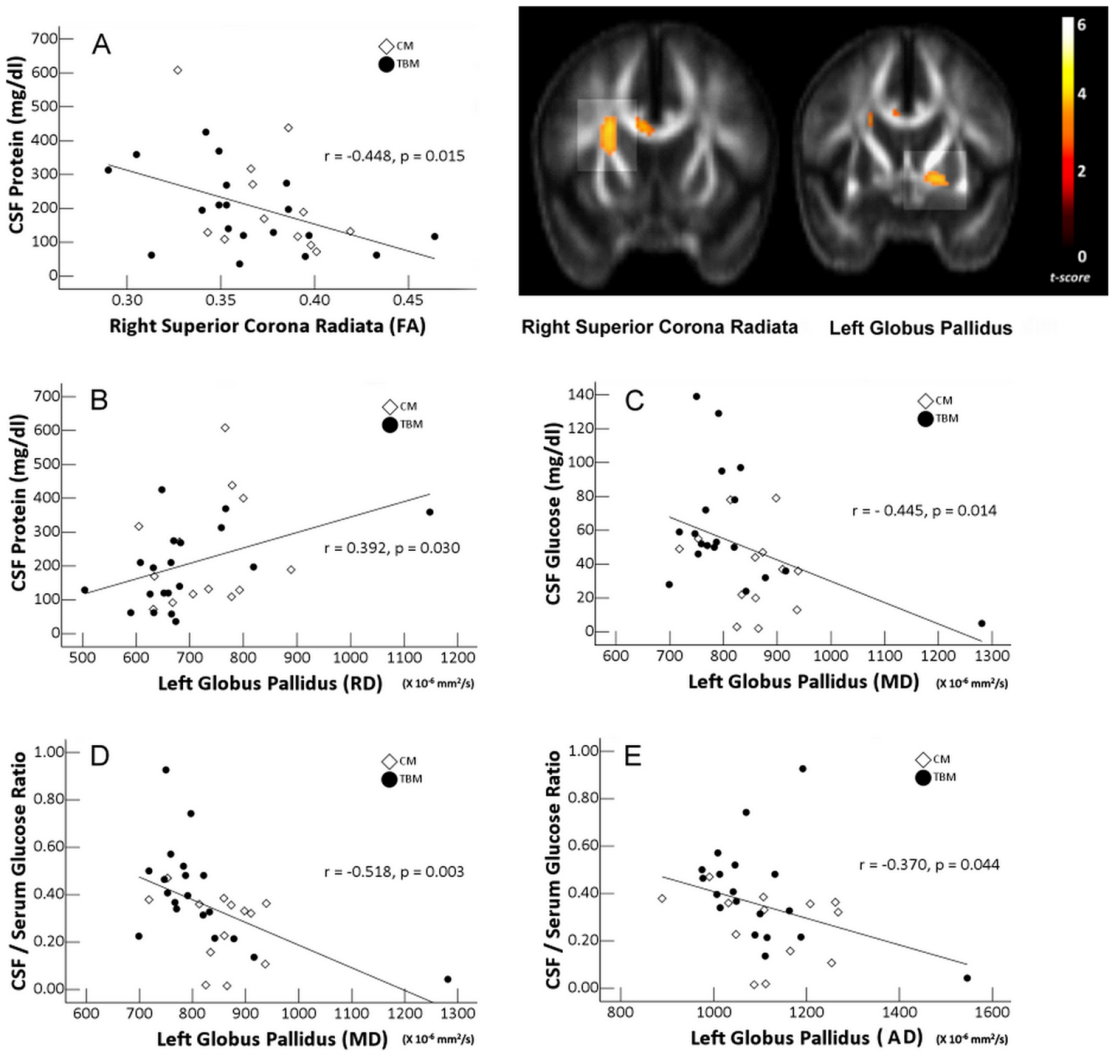
Correlations between CSF profile of chronic meningitis and DTI indices after adjustments for covariates. The right cingulate gyrus (BA 32, superior corona radiata) and the WM close to left globus pallidus are shown.

Partial correlation analyses revealed that higher protein level was associated with decreased FA in the right cingulate gyrus (BA 32, superior corona radiate) and increased RD in the WM close to the left globus pallidus (Fig. 3). Moreover, lower CSF glucose level and lower CSF/serum glucose ratio were associated with increased MD in the WM close to the globus pallidus. Lower CSF/serum glucose ratio was also associated with increased AD in the WM near the globus pallidus.

#### Relationship between FA and NP Tests

There were significant correlations between worse attention function (*orientation*) and decreased FA value of the para-hippocampus (right inferior longitudinal fasciculus). Worse executive function (*digit symbol coding*, *similarity, picture arrange,* and *information and comprehension*) was associated with decreased FA of the bilateral para-hippocampus (bilateral inferior longitudinal fasciculus), cingulate gyrus (right cingulum and superior corona radiata), and the WM near the left globus pallidus. Worse visuo-construction function (*picture complete* and *block design*) correlated with decreased FA of the bilateral para-hippocampus (bilateral inferior longitudinal fasciculus) (Table 3).

**Table 3:** Correlation between diffusion tensor imaging (DTI) abnormalities and cognitive function after adjustments for age, sex, and education in HIV-negative chronic meningitis.

NP Test	DTI Metrics	Anatomical Lobe	Brodmann Area	White Matter Tract	Correlation (r)	*p* value
***Attention Function***						
*Digit Span*	MD	Cingulate Gyrus	32	R Superior Corona Radiata	−0.266	0.044
*Orientation*	FA	Para-hippocampal Gyrus	19	R Inferior Longitudinal Fasciculus	0.365	0.004
	AD	Para-hippocampal Gyrus	19	L Inferior Longitudinal Fasciculus	−0.450	<0.001
	AD	Cingulate Gyrus	32	R Superior Corona Radiata	−0.428	0.001
	RD	Para-hippocampal Gyrus	19	L Inferior Longitudinal Fasciculus	−0.529	<0.001
	RD	Cingulate Gyrus	32	R Superior Corona Radiata	−0.331	0.010
	RD	Para-hippocampal Gyrus	19	R Inferior Longitudinal Fasciculus	−0.454	<0.001
	MD	Para-hippocampal Gyrus	19	L Inferior Longitudinal Fasciculus	−0.543	0.000
	MD	Cingulate Gyrus	32	R Superior Corona Radiata	−0.380	0.003
	MD	Para-hippocampal Gyrus	19	R Inferior Longitudinal Fasciculus	−0.434	0.001
***Executive Function***						
*Digit Symbol Coding*	*FA*	Para-hippocampal Gyrus	19	L Inferior Longitudinal Fasciculus	0.275	0.040
	*RD*	Lentiform Nucleus		L WM close to Globus Pallidus	−0.413	0.002
	*MD*	Lentiform Nucleus		L WM close to Globus Pallidus	−0.370	0.005
*Similarity*	*FA*	Para-hippocampal Gyrus	19	L Inferior Longitudinal Fasciculus	0.343	0.009
	*AD*	Cingulate Gyrus	24	R Cingulum	−0.362	0.006
	*AD*	Cingulate Gyrus	32	R Superior Corona Radiata	−0.416	0.001
	*AD*	Precentral Gyrus	6	L Superior Longitudinal Fasciculus	−0.268	0.044
	*AD*	Cingulate Gyrus	24	L Superior Corona Radiata	−0.337	0.010
	*RD*	Cingulate Gyrus	24	R Cingulum	−0.361	0.006
	*RD*	Cingulate Gyrus	32	R Superior Corona Radiata	−0.418	0.001
	*RD*	Cingulate Gyrus	24	L Superior Corona Radiata	−0.364	0.005
	*MD*	Cingulate Gyrus	24	R Cingulum	−0.375	0.004
	*MD*	Cingulate Gyrus	32	R Superior Corona Radiata	−0.432	0.001
	*MD*	Cingulate Gyrus	24	L Superior Corona Radiata	−0.356	0.006
*Picture Arrangement*	*FA*	Para-hippocampal Gyrus	19	R Inferior Longitudinal Fasciculus	0.290	0.030
	*FA*	Lentiform Nucleus		L WM close to Globus Pallidus	0.298	0.026
*Information*	*FA*	Para-hippocampal Gyrus	19	L Inferior Longitudinal Fasciculus	0.287	0.028
	*FA*	Cingulate Gyrus	24	R Cingulum	0.260	0.047
	*FA*	Cingulate Gyrus	32	R Superior Corona Radiata	0.282	0.031
	*FA*	Lentiform Nucleus		L WM close to Globus Pallidus	0.319	0.014
	*RD*	Cingulate Gyrus	32	R Superior Corona Radiata	−0.332	0.010
	*RD*	Cingulate Gyrus	24	L Superior Corona Radiata	−0.268	0.040
	*MD*	Cingulate Gyrus	32	R Superior Corona Radiata	−0.313	0.016
*Comprehension*	*FA*	Cingulate Gyrus	24	R Cingulum	0.279	0.033
	*AD*	Cingulate Gyrus	32	R Superior Corona Radiata	−0.429	0.001
	*AD*	Cingulate Gyrus	24	L Superior Corona Radiata	−0.322	0.013
	*RD*	Cingulate Gyrus	24	R Cingulum	−0.285	0.029
	*RD*	Cingulate Gyrus	32	R Superior Corona Radiata	−0.393	0.002
	*RD*	Cingulate Gyrus	24	L Superior Corona Radiata	−0.328	0.011
	*MD*	Cingulate Gyrus	32	R Superior Corona Radiata	−0.421	0.001
	*MD*	Cingulate Gyrus	24	L Superior Corona Radiata	−0.329	0.011
***Visuo-Construction Function***						
*Picture Complete*	*FA*	Para-hippocampal Gyrus	19	R Inferior Longitudinal Fasciculus	0.295	0.026
*Block Design*	*FA*	Para-hippocampal Gyrus	19	L Inferior Longitudinal Fasciculus	0.311	0.019
	*FA*	Para-hippocampal Gyrus	19	R Inferior Longitudinal Fasciculus	0.302	0.023
	*RD*	Para-hippocampal Gyrus	19	R Inferior Longitudinal Fasciculus	−0.285	0.032
	*RD*	Lentiform Nucleus		L WM close to Globus Pallidus	−0.309	0.019

#### Relationship between MD and NP Tests

There were significant correlations between worse attention function (*digit span*, and *orientation*) and increased MD value of the cingulate gyrus (right superior corona radiate) and bilateral para-hippocampus (bilateral inferior longitudinal fasciculus). Worse executive function (*digit symbol coding*, *similarity, information and comprehension*) was associated with increased MD value of the WM close to the left globus pallidus and bilateral cingulate gyrus (right cingulum and bilateral corona radiata).

#### Relationship between AD and NP Tests

There were significant correlations between worse attention function (*orientation*) and decreased FA value of the right para-hippocampal gyrus (right inferior longitudinal fasciculus) and cingulate gyrus (right superior corona radiate). Worse executive function (s*imilarity* and *comprehension*) was associated with increased AD in the bilateral cingulate gyrus (right cingulum and bilateral corona radiata) and left pre-central gyrus (superior longitudinal fasciculus).

#### Relationship between RD and NP Tests

There were significant correlations between worse attention function (*orientation*) and decreased FA value of the para-hippocampal gyrus (bilateral right inferior longitudinal fasciculus) and cingulate gyrus (right superior corona radiata). Worse executive function (*digit symbol coding*, *similarity*, and *information and comprehension*) was associated with increased RD of the WM close to the left globus pallidus and bilateral cingulate gyrus (right cingulum and bilateral corona radiate). Worse visuo-construction function (*block design*) correlated with increased RD of the right para-hippocampal gyrus (inferior longitudinal fasciculus) and WM near the left globus pallidus.

## Discussion

In the present study, HIV-negative chronic meningitis has heterogeneous changes in micro-structures in the limbic system and WM close to the globus pallidus, as revealed by multiple diffusion tensor metrics (FA, MD, AD, and RD). These changes are associated with the initial disease severity. There are attention, execution, memory, speech, language, and visuo-construction function deficits in chronic meningitis that are rarely reported. Different limbic system deficits are also significantly correlated to scores on these tests, revealing a direct relationship between impaired cognitive functions and micro-structure abnormalities.

There is growing evidence, both experimentally and epidemiologically, of the association of neuro-infection with cerebral WM injury [25]. In histological study, abundantly infiltration of *Cryptococcus neoformans* with prominent immune reaction were revealed in cerebral WM and cortex adjacent to the leptomeninges, but these findings were not observed in the subcortical and cortical lesions [26]. Distinct WM disruption profiles demonstrate heterogeneous pathologic processes affecting different WM areas in chronic meningitis. Different alterations in WM integrity may be the end result of interactions among (1) acute stage inflammation, (2) ischemia after vessel occlusion, (3) pressure stress from hydrocephalus, and (4) brain tissue vulnerabilities [13], [27]. Their contributions to DTI changes are not be fully resolved in this cross-sectional analysis.

The FA is derived from directional diffusivities of diffusion tensor imaging. Unfortunately, FA is a highly non-specific marker. Consideration of the different directional diffusivities is important because if changes in diffusion along the axial direction are proportional to those along the radial directions, then FA (which is a function of the ratio) will remain unchanged [28]. Decreased FA can correspond to different pathologic findings like demyelination/dysmyelination, axonal loss, gliosis, and tissue inflammation [29]. This decrease occurs after either an increase in RD or a decrease in AD, or both. Increased RD reflects increased water diffusion in the perpendicular direction.

In the present study, decreased FA, increased RD, and a much smaller or no change in AD such as the para-hippocampus, cingulate gyrus, pre-central gyrus, and WM close to globus pallidus (Table 2), have been suggested as myelin injury [16]. However, decreased FA with reduced AD but increased RD in the left cuneus (forceps major) may likewise be associated with axonal damage and demyelination or the fiber re-organization found in neuro-degenerative diseases [30].

Increased MD reflects isotropic diffusion with the free movement of water that is commonly observed in gliosis after infarction. In chronic meningitis, 30–50% of patients reportedly experience cerebral infarction [2]. In TBM, the rich focus ruptures into the sub-arachnoid space, causing meningitis. A thick, gelatinous exudate infiltrates the cortical or meningeal blood vessels, producing obliterative vasculitis or infarction. In CM, inflammation is initially confined to the sub-arachnoid space and is prone to infiltrating the Virchow-Robin peri-vascular spaces which tend to exhibit a *soap bubble* appearance by MRI [31]. Thrombo-embolism from vasculitis within these small peri-vascular spaces further leads to tissue ischemia via decreased cerebral perfusion.

In the present study, the significantly decreased FA and increase MD in the right para-hippocampal gyrus (BA 19) and right cingulate gyrus (BA 32) may indicate the most severe WM ischemia, with subsequent gliosis [29]. Thirteen of 32 patients present with either basal ganglia infarction or prominent peri-vascular space. However, decreased FA in the WM close to the globus pallidus seem to primarily come from myelin injury (increased RD but no change in MD), instead of gliosis. More sensitive DTI for detecting WM than gray matter ischemia may explain this phenomenon [32]. The true mechanism is unknown.

Hydrocephalus is the most common serious complication of chronic meningitis and is strongly associated with long-term outcome [3], [33]. In chronic meningitis, basal meningitis eventually leads to obstructive hydrocephalus from obstruction of the basilar cisterns. In chronic hydrocephalus, WM loss is associated with deficits in motor and cognitive functions in a previous animal and human study [34]. In the present study, susceptible anatomies with DTI differences between groups are consistent findings of a previous study and suggest that mechanical injury from hydrocephalus may be part of the pathophysiology of WM injury. However, most differential limbic system DTI changes result from increased RD value, which points more to myelin loss. The findings here are similar to another histologic study in neonatal meningitis where there is myelin loss in the peri-ventricular region showing infiltration with glial cells [35]. However, it is also possible that the decreased FA and increased RD may indirectly represent loss of axial diffusivity or axonal injury, although this is difficult to substantiate given the absence of statistical significance. To date, no conclusive findings on meningitis can be drawn from DTI studies.

Malik et al. report that FA values decrease without information about AD and RD in the peri-ventricular WM in the sub-acute stage of neonatal meningitis, even in patients with normal outcomes [13]. In the acute stage, hydrocephalus can compress the WM with increased AD and decreased RD, resulting in an overall increase in FA value in bacterial meningitis [36]. Alterations in DTI indices may be associated with a combination of stretch injury, impaired blood circulation, and accumulated damage from pathogens/waste products in the CSF [37].

Regardless of TBM or CM, CSF parameters like high CSF lactate and protein levels, and low CSF glucose are identified as poor prognostic indicators [38], [39]. The association between worse CSF profile in the acute stage and long-term decline in DTI indices establish the possible etiology of their respective chronic neuro-psychiatric sequelae. Results of the present study are consistent with previous arguments that the initial stage of the disease on presentation is a major prognostic indicator of morbidity and mortality [40], [41]. Poor neurologic outcomes can be predicted based the presenting stage of the disease [40]–[42]. The high microbial burden in the sub-arachnoid space can damage vessels in the circle of Willis and affect the globus pallidus more directly than others. The results can explain the pathogenesis of chronic meningitis.

Extensive impairment of neuro-psychological performance and its significant correlation with WM damage in the limbic system underscore the pathologic role of the limbic system in chronic meningitis. The dorsal part of the anterior cingulate cortex (ACC) (BA 24 and 32) is a central station for processing top-down and bottom-up stimuli and for assigning appropriate control to other brain cortices. The results here are consistent with previous reports that ACC (BA 24 and 32) abnormalities in morphology and histopathology constitute impaired executive and attention functions in multiple neurologic and psychiatric disorders [43], [44]. The consistent damage in ACC in meningitis, chronic hydrocephalus in animals and humans, and in the present study may make ACC a specific pilot anatomy to help improve long-term outcomes in chronic meningitis and guide future interventional treatment strategies.

Abnormal DTI findings in the WM near the globus pallidus also partly contribute to the neuro-psychiatric deficits in chronic meningitis. There is growing evidence that the globus pallidus is one of the neural substrates of executive cognitive function [45] and that it plays an important role in the cortico – striatal – pallidal – thalamo – cortical loops [46]. Its function is to serve as a limbic-somatic motor interface in the planning and inhibition of movements. The para-hippocampus (BA 19) is a histologically delineated band antero-laterally abutting the visual area 18 and is responsible for the heterogeneous visual information collection, including feature-extracting, shape recognition, attention, and multi-modal integrating functions [47]. Together with the cingulate gyrus and globus pallidus, the para-hippocampus reveals an association between declined DTI indices and the relative decline in attention, execution, and visuo-construction functions.

The interpretation of the findings here must be tempered by some limitations. The results are based on a relatively small cohort for both TBM and CM. The study did not enroll patients with severe sequelae. Thus, there is uncertainty in assessing the DTI findings of critically-ill patients and those with poor prognosis. Second, the DTI findings may be influenced by the duration of hydrocephalus, increased intracranial pressure, antimicrobial therapy, and follow-up period. These may be also influence by other drugs (e.g. steroids and hyper-osmolarity agents) that are commonly used in patients with meningitis. These drugs may cause potential bias in the interpretation of DTI findings. Moreover, further studies to determine whether the observed effects are due to pre-existing neurologic or psychiatric illnesses and micro-angiopathy in elderly subjects after long-term follow-up are warranted as these may be unrelated to the disease process. Lastly, a cross-sectional study must be performed on two sample groups rather than a longitudinal study following treatment because when the WM damage begins remains unsolved. Recurrent infection is also an important risk factor for progressive white matter injury [48].

In conclusion, there are significant WM differences in the limbic system-associated anatomy and the WM close to the globus pallidus between chronic meningitis and healthy controls. After exploring the possible pathophysiology and psychopathology with initial disease severity and long-term neuro-psychiatric sequelae, abnormalities in the ACC, para-hippocampus, and globus pallidus may be specific biomarkers for disease evaluation. A longitudinal study combined with evaluation of the timing of medical and intervention therapy is warranted.

## Supporting Information

Table S1 Comparison of fractional anisotropy (FA) values between tuberculous meningitis (TBM) and controls, between cryptococcal meningitis (CM) and controls, and between TBM and CM
(DOC)
