# Supplementary material for: Diffusion Tensor Imaging Study of White Matter Damage in Chronic Meningitis
Source: PLoS One. 2014 Jun 3;9(6):e98210. doi: 10.1371/journal.pone.0098210 (PMC4043527; doi:10.1371/journal.pone.0098210)
Supplement: Table S1 — Comparison of fractional anisotropy (FA) values between tuberculous meningitis (TBM) and controls, between cryptococcal meningitis (CM) and controls, and between TBM and CM (DOC) [file pone.0098210.s001.doc]

**Supplementary Table S1. Comparison of fractional anisotropy (FA) values between tuberculous meningitis (TBM) and controls, between cryptococcal meningitis (CM) and controls, and between TBM and CM**

| MNI atlas | | | Voxel size | White matter tract | Nearest Grey Matter^Ψ^ | FA mean (SD) | | *t_max_* | Diffusivity values | | |
| --- | --- | --- | --- | --- | --- | --- | --- | --- | --- | --- | --- |
| coordinates | | |  |  |  |  |  |  |  |  |  |
| X | Y | Z |  |  |  |  |  |  | MD | AD | RD |
| Decreased FA in TBM than NC | | | | |  | NC | TBM |  | TBM-NC | | |
| 4 | -22 | 74 | 26 | R Cortico-spinal Tract | Para-central Lobule, BA6 | 0.28 (0.07) | 0.22 (0.07) | 4.01 | 150.6 | 109.1 | 171.3 |
| 20 | -64 | 0 | 29 | R Inferior Longitudinal Fasciculus | Para-hippocampal Gyrus, BA19 | 0.35 (0.07) | 0.28 (0.08) | 3.89 | 34.1 | -24.8 | 63.6 |
| 8 | 14 | 28 | 42 | R Cingulum | Cingulate Gyrus, BA24 | 0.61 (0.09) | 0.48 (0.16) | 3.82 | 302.1 | 189.6 | 358.4 |
| -34 | -20 | 66 | 27 | L Cortico-spinal Tract | Pre-central Gyrus, BA4 | 0.32 (0.09) | 0.23 (0.10) | 3.70 | 297.5 | 263.9 | 314.4 |
| -18 | -12 | 40 | 20 | L Superior Corona Radiata | Cingulate Gyrus, BA24 | 0.38 (0.08) | 0.32 (0.06) | 3.60 | 221.6 | 206.9 | 228.9 |
| 26 | 14 | 24 | 21 | R Anterior Corona Radiata |  | 0.40 (0.05) | 0.35 (0.05) | 3.45 | 104.7 | 91.4 | 111.4 |
| Decreased FA in CM than NC | | | | |  | NC | CM |  | CM-NC | | |
| -22 | -70 | 16 | 88 | L Forceps Major | Cuneus, BA 30 | 0.43 (0.08) | 0.28 (0.11) | 4.28 | 75.2 | -88.9 | 157.4 |
| -18 | 2 | -4 | 48 | L WM close to Globus Pallidus | Lentiform Nucleus | 0.37 (0.06) | 0.31 (0.05) | 3.69 | 69.8 | 22.3 | 94.6 |
| Decreased FA in TBM than CM | | | | |  | CM | TBM |  | TBM-CM | | |
| 30 | -16 | 72 | 132 | R Cortico-spinal Tract | Pre-central Gyrus, BA4 | 0.41 (0.04) | 0.33 (0.06) | 3.87 | 100.3 | 15.4 | 142.8 |
| -38 | 12 | 18 | 29 | L Superior Longitudinal Fasciculus | Insula, BA13 | 0.45 (0.06) | 0.35 (0.08) | 3.58 | 32.0 | -75.9 | 85.9 |
| 16 | -58 | -62 | 74 | R Inferior Cerebellar Peduncle |  | 0.32 (0.03) | 0.27 (0.03) | 3.55 | -6.7 | -54.8 | 17.3 |
| 20 | -32 | 68 | 49 | R Cortico-spinal Tract | Post-central Gyrus, BA3 | 0.37 (0.07) | 0.28 (0.09) | 3.50 | 122.6 | 31.8 | 168.1 |
| -44 | -58 | -8 | 30 | L Inferior Longitudinal Fasciculus | Fusiform Gyrus, BA37 | 0.28 (0.06) | 0.20 (0.07) | 3.49 | 46.7 | -32.5 | 86.4 |
| 32 | 32 | 12 | 47 | R Inferior Fronto-occipital Fasciculus | Anterior Cingulate, BA24 | 0.37 (0.08) | 0.30 (0.06) | 3.39 | 18.0 | -45.1 | 49.6 |
| 42 | -50 | 54 | 48 | R Superior Longitudinal Fasciculus | Superior Parietal Lobule, BA7 | 0.29 (0.12) | 0.19 (0.06) | 3.39 | 111.4 | 9.6 | 162.3 |
| -22 | 0 | 68 | 20 | L Superior Corona Radiata | Superior Frontal Gyrus, BA6 | 0.29 (0.12) | 0.19 (0.07) | 3.22 | 183.5 | 94.4 | 228.1 |
| -30 | -76 | -12 | 43 | L Inferior Longitudinal Fasciculus | Declive | 0.29 (0.06) | 0.21 (0.06) | 3.18 | 100.2 | 45.8 | 127.5 |
| Decreased FA in CM than TBM | | | | |  | TBM | CM |  | CM-TBM | | |
| 6 | 12 | -18 | 29 | R Uncinate Fasciculus | Anterior Cingulate, BA24 | 0.24 (0.04) | 0.20 (0.05) | 3.77 | 34.3 | -6.9 | 54.9 |
| 48 | 18 | -16 | 25 | R Inferior Longitudinal Fasciculus | Inferior Frontal Gyrus, BA47 | 0.23 (0.05) | 0.22 (0.03) | 3.06 | -90.1 | -124 | -72.9 |
| 24 | -62 | 16 | 25 | R Forceps Major | Posterior Cingulate, BA31 | 0.36 (0.18) | 0.22 (0.10) | 3.04 | 115.9 | -37.6 | 192.7 |
| -46 | 10 | -22 | 27 | L Inferior Longitudinal Fasciculus | Superior Temporal Gyrus, BA38 | 0.26 (0.05) | 0.21 (0.05) | 3.03 | 283.1 | 258.3 | 295.4 |

The diffusivity values describe differences (meningitis vs. normal) in mean diffusivity (MD), axial diffusivity (AD), and radial diffusivities (RD) (mm^2^/s) multiplied by 10^-6^

*Significant differences among MD, AD, and RD were adjusted with age, sex and education as covariates (*p*≤0.05).

^Ψ^Nearest Gray Matter was near the center of the 5-mm radius search area.

Abbreviations: MNI, Montreal Neurological Institute; BA, Brodmann area
